# Retraction: Detection of Histone Acetylation Levels in the Dorsal Hippocampus Reveals Early Tagging on Specific Residues of H2B and H4 Histones in Response to Learning

**DOI:** 10.1371/journal.pone.0296474

**Published:** 2023-12-22

**Authors:** The PLOS ONE Editors

After this article [1] was published, concerns were raised about some of the western blots in Figures 1 and 2. Specifically:

- In Figure 1B, there are vertical discontinuities between:
  - Lanes 2 and 3, and 5 and 6 in the K5Ac panel.
  - Lanes 2 and 3, 4 and 5, and 5 and 6 in the Tetra Ac panel.
  - Lanes 2 and 3 in the K12Ac panel.
  - Lanes 2 and 3 in the total H2B panel.
- In Figure 2C, there are vertical discontinuities between:
  - Lanes 1 and 2 in the K12Ac panel.
  - Lanes 2 and 3 in the Tetra Ac panel.
  - Lanes 2 and 3, and 6 and 7 in the total H2B panel.
  - Lanes 2 and 3, and 3 and 4 in the K9K14Ac panel.
  - Lanes 2 and 3, 3 and 4, and 6 and 7 in the total H3 panel.

During editorial follow up on these issues, the authors stated that the data underlying this article [1] are no longer available.

The corresponding author stated that images were spliced because only a representative subset of samples (lanes) from the original experiments were included in the published figures. The ninth author stated that the data presented in Figures 1B and 2C in [1] were provided as illustrations, and that the semi-quantitative data reported in [1] correspond to evaluations referencing each assessment to the control from the same experimental sample. The captions for Figures 1B and 2C in [1] state that ‘Typical western blots are presented/shown in duplicates’.

The corresponding author also stated that total histones were not run on the same gel as modified histones as they migrate to the same position on the gel, but they indicated all quantifications were performed using the appropriate corresponding control samples.

In the absence of the original raw image data, the above concerns cannot be resolved and PLOS cannot verify the reliability of the reported results and conclusions. Therefore, the *PLOS ONE* Editors retract this article.

ALB, MM, AB, APdV, OB, JCC, RN, and MAM did not agree with the retraction. AS and JPL either did not respond directly or could not be reached.

ALB stands by the article’s findings.
